# Efficacy and Safety of Electroacupuncture for Postoperative Insomnia in Patients With Spinal Metastasis: Protocol of a Prospective, Multicenter, Randomized Controlled Study

**DOI:** 10.2196/81489

**Published:** 2026-06-08

**Authors:** Cuifang Jiang, Mengchen Yin, Xia Peng, Junqiao Zhao, Fan Zhang, Junming Ma, Dong Wang, Yan Cao

**Affiliations:** 1 Department of Acupuncture and Moxibustion, Shanghai Municipal Hospital of Traditional Chinese Medicine, Shanghai University of Traditional Chinese Medicine, No. 274 Zhijiang Middle Road, Jing'an District, Shanghai, 200071, China, 86 1-502-131-9564; 2 Department of Spine, Yueyang Hospital of Integrated Traditional Chinese and Western Medicine, Shanghai University of Traditional Chinese Medicine, Shanghai, China; 3 Department of Encephalopathy, Shanghai Jiading Hospital of Traditional Chinese Medicine, Shanghai, China; 4 Department of Nephrology, Department of Orthopedics, Longhua Hospital, Shanghai University of Traditional Chinese Medicine, Shanghai, China; 5 Department of Orthopedics, Hangzhou TCM Hospital Zhejiang Chinese Medical University, Hangzhou, China

**Keywords:** electroacupuncture, spinal metastasis, postoperative insomnia, randomized controlled trial, clinical protocol, sleep quality

## Abstract

**Background:**

Postoperative insomnia is one of the common complaints caused by spinal metastatic cancer surgery. It affects patients’ functional recovery, greatly reduces their quality of life, and adversely impacts disease prognosis. Compared with traditional pharmacological treatments, acupuncture is an alternative therapy for postoperative insomnia. However, standardized, high-quality randomized controlled trials on electroacupuncture for postoperative insomnia in patients with spinal metastasis (SM) are scarce, and there is a lack of clear inclusion criteria for this specific population. Postoperative insomnia in patients with SM has distinct clinical characteristics compared with general cancer-related insomnia, necessitating targeted investigation.

**Objective:**

This study aims to evaluate the efficacy and safety of electroacupuncture in the treatment of postoperative insomnia in patients with SM, and to provide high-level clinical evidence for the inclusion of electroacupuncture in the clinical management plan of postoperative insomnia in patients with SM.

**Methods:**

This is a study protocol for a randomized controlled trial. We will randomly assign 196 patients with insomnia after spinal metastatic cancer surgery to the acupuncture group (n=98) or the control group (sham acupuncture group; n=98). All participants will be treated on the first day after surgery and receive 12 sessions in total (30 min per session, 3 sessions per wk for 4 weeks). The primary outcome is the change in Pittsburgh Sleep Quality Index score from baseline to post treatment (wk 4). The secondary outcomes include actigraphy records (sleep efficiency, number of sleep awakenings, total sleep time, sleep latency, and wake after sleep onset), Insomnia Severity Index, Spine Oncology Study Group Outcomes Questionnaire 2.0, and Patient Health Questionnaire-9. All results will adhere to the intention-to-treat principle and will be evaluated at baseline, posttreatment (wk 4), and follow-up (wk 12).

**Results:**

This study was funded in June 2023 (supported by the Project of Shanghai Municipal Health Commission, National Natural Science Foundation of China, etc). Recruitment will start in mid-2026 and end in December 2027. Data collection will be completed in October 2027, and data analysis is expected to be finished in December 2027. The results of this study are anticipated to be published in the first half of 2028.

**Conclusions:**

This study is designed to rigorously assess the therapeutic value of electroacupuncture for postoperative insomnia in patients with SM. If proven effective, electroacupuncture is expected to become a safe and feasible alternative or complementary therapy for this population, reducing reliance on hypnotic drugs and improving patients’ quality of life and prognosis. The results will fill the gap in current clinical evidence for electroacupuncture in the treatment of spinal metastatic postoperative insomnia and provide a basis for the optimization of clinical treatment strategies.

## Introduction

Spinal metastasis (SM) is a common complication in the advanced stages of malignant tumors, with reported prevalence ranging from approximately 40% to 70% among patients with systemic cancer. SM frequently leads to vertebral destruction, spinal instability, and neural element compression, resulting in pain, neurological dysfunction, impaired motor function, and, in severe cases, paralysis [1-4].

As many as 26% of patients with cancer suffer from insomnia, and postoperative rehabilitation and care for these patients is a heavy social burden. Insomnia is a common consequence after SM surgery, mainly manifesting as difficulty falling asleep, easy awakening, insufficient sleep time, and poor sleep quality. Patients often feel fatigued after waking [5,6]. Studies have shown that insomnia not only affects patients’ functional recovery, daily living ability, quality of life, physical and mental health, and neurological rehabilitation but also impacts cancer prognosis. It can lead to decreased immune function in patients with cancer, increased expression of cancer-promoting factors, and higher mortality [7,8]. In addition, survivors of cancer with persistent insomnia frequently present with comorbid anxiety and depressive symptoms, which, if left untreated, may further compromise immune function, reduce survival, and substantially diminish overall quality of life [9,10].

Numerous randomized controlled trials (RCTs) have evaluated the efficacy and safety of electroacupuncture for cancer-related insomnia (CRI), but SM postoperative insomnia has unique characteristics that distinguish it from general CRI. First, the pathogenesis is more complex; SM surgery involves spinal structure reconstruction, which may cause persistent postoperative pain, limited spinal movement, and neurological irritation, all of which directly interfere with sleep initiation and maintenance. In contrast, general CRI is more often associated with systemic symptoms such as tumor-related fatigue, anxiety, and drug side effects. Second, the sleep disturbance pattern is specific; patients with postoperative SM often experience sleep fragmentation due to nocturnal pain and positional restrictions, while general CRI is more likely to present as difficulty falling asleep or early awakening. Third, the rehabilitation context differs; patients with SM are in a critical period of spinal function recovery, and sleep quality directly affects tissue repair and neurological function restoration, whereas general CRI has a more indirect impact on disease progression. These unique characteristics indicate that the efficacy and mechanism of electroacupuncture for SM postoperative insomnia may differ from those for general CRI, justifying the necessity of this targeted study.

Studies in recent years have further confirmed the multifaceted adverse impact of postoperative insomnia on patients with SM. In terms of physical recovery, insomnia can reduce the body’s immune function, inhibit the repair of damaged tissues, and delay the recovery of postoperative motor and neurological functions [11-13]. In terms of disease prognosis, insomnia can up-regulate the expression of cancer-promoting factors (such as IL-6 and TNF-α), increase the risk of tumor recurrence and metastasis, and reduce the overall survival rate of patients [14-16]. In terms of mental health, long-term insomnia is closely associated with anxiety, depression, and other negative emotions, which further aggravates the decline in quality of life [17,18]. A 2023 study by Kim et al [8] found that CRI can mediate the impact of sleep disturbance on disease progression fear in patients with postoperative lung cancer, highlighting the importance of intervening in insomnia.

At present, the clinical treatment of postoperative insomnia mainly relies on pharmacological methods. Commonly used drugs include benzodiazepines, new nonbenzodiazepines, and antidepressants [19]. Although these drugs can temporarily improve sleep symptoms, long-term use is prone to tolerance, addiction, cognitive impairment, and other adverse reactions [20,21], making them unsuitable for patients with SM who need long-term rehabilitation. Therefore, exploring safe and effective nonpharmacological therapies has become an urgent clinical need.

As a nonpharmacological treatment of Traditional Chinese Medicine, acupuncture exerts therapeutic effects on insomnia by inserting needles at specific body points. A study found that acupuncture is effective and safe in treating insomnia, worthy of clinical promotion, and may be a promising nonpharmacological alternative therapy [22]. Due to its efficacy and safety, electroacupuncture is being used to treat insomnia and relieve cancer-related symptoms [23]. Electroacupuncture, as an important part of Traditional Chinese Medicine, acts on specific acupoints of the human body through acupuncture needles and electrical stimulation, regulating the body’s functional state to soothe the nerves and improve sleep. A meta-analysis by Zhao et al [22] confirmed that acupuncture is effective and safe for primary insomnia, significantly improving patients’ sleep quality [24]. Another systematic review by Guo et al [25] pointed out that acupuncture-related therapies have potential advantages in the treatment of CRI, but the quality of evidence is limited. However, most existing studies focus on general CRI, lacking targeted research on SM postoperative insomnia. Patients with SM have unique clinical characteristics such as postoperative pain, spinal instability, and neurological damage, which may affect the efficacy of electroacupuncture. In addition, existing studies have problems, such as small sample sizes, single-center designs, and unclear inclusion criteria, resulting in insufficient reliability and generalizability of results.

This study aims to fill the gap in existing research by conducting a prospective, multicenter, RCT. The specific research hypotheses are (1) electroacupuncture can significantly improve the sleep quality of patients with postoperative insomnia after SM, as reflected by the reduction of Pittsburgh Sleep Quality Index (PSQI) score; (2) electroacupuncture can improve sleep-related objective indicators (sleep efficiency, total sleep time [TST], etc), reduce the severity of insomnia, improve quality of life, and alleviate depressive symptoms; (3) electroacupuncture is safe for patients with postoperative SM, with a low incidence of adverse events (AEs). Through this study, we expect to provide high-level clinical evidence for the application of electroacupuncture in the treatment of SM postoperative insomnia and offer a new treatment option for clinical practice.

## Methods

### Study Design and Setting

This will be a 16-week prospective, multicenter RCT carried out at 4 clinical institutions, including (1) Longhua Hospital, Shanghai University of Traditional Chinese Medicine; (2) Shanghai Municipal Hospital of Traditional Chinese Medicine, Shanghai University of Traditional Chinese Medicine; (3) Hangzhou TCM Hospital, Zhejiang Chinese Medical University; (4) Shanghai Tonghe Orthopedic Hospital; and (5) Department of Spine, Yueyang Hospital of Integrated Traditional Chinese and Western Medicine, Shanghai University of Traditional Chinese Medicine, Shanghai (200437), China.

The 16-week study period (4-wk treatment+8 wk follow-up) is determined based on the characteristics of postoperative recovery and the therapeutic cycle of electroacupuncture. The postoperative recovery of patients with SM is a long process, and sleep disturbance may persist for several months. A 4-week treatment cycle is consistent with the conventional course of acupuncture for insomnia, facilitating the observation of immediate therapeutic effects [26]. The 8-week follow-up period can further evaluate the sustainability of the therapeutic effect and whether insomnia symptoms rebound, which is of great significance for guiding long-term clinical management. Previous studies have shown that the therapeutic effect of electroacupuncture on insomnia can be maintained for 1‐3 months after treatment, so an 8-week follow-up is set to fully observe the long-term effect [27,28].

The schedule of treatment assessments and data collection is presented in Table 1. The trial protocol was developed in accordance with the SPIRIT (Standard Protocol Items: Recommendations for Interventional Trials) 2013 guidelines, and the Standards for Reporting Interventions in Clinical Trials of Acupuncture (STRICTA) to ensure methodological rigor and transparency of the intervention reporting [29,30]. There is a deviation in recruitment timeline. The initial recruitment start date recorded in the trial registry was May 2025. Owing to logistical preparation and multicenter institutional coordination for the study, the recruitment timeline has been revised and the confirmed start date is March 2026, which is the actual operational schedule for this trial.

**Table 1.** Schedule of enrollment, interventions, and assessments.

Study period	Time point (wk)							
	Enrollment		Intervention				Follow-up period	
	Week –1	Week 0	Week 1	Week 2	Week 3	Week 4	Week 8	Week 16
Eligibility screening	✓							
Informed consent	✓							
Medical history	✓							
Randomization		✓						
Intervention								
Electroacupuncture			✓	✓	✓	✓		
Sham electroacupuncture			✓	✓	✓	✓		
Primary outcomes								
PSQI^a^		✓				✓	✓	✓
Secondary outcomes								
Actigraphy		✓				✓	✓	✓
ISI^b^		✓				✓	✓	✓
SOSGOQ 2.0^c^		✓				✓	✓	✓
PHQ-9^d^		✓				✓	✓	✓
Other assessment								
Safety of electroacupuncture			✓	✓	✓	✓		
Adverse events			✓	✓	✓	✓	✓	✓
Assessment of credibility		✓						
Assessment of blinding success						✓		

a PSQI: Pittsburgh Sleep Quality Index.

b ISI: Insomnia Severity Index.

c SOSGOQ 2.0: Spine Oncology Study Group Outcomes Questionnaire 2.0.

d PHQ-9: Patient Health Questionnaire-9.

### Ethical Considerations

#### Ethics Committee Approval

The study protocol and related documents have been approved by the Ethics Review Committee of Shanghai Municipal Hospital of Traditional Chinese Medicine (approval 2025SHL-KY-70‐01), and have been filed with the ethics committees of the other 4 participating centers.

#### Informed Consent

Before screening, researchers will fully inform potential participants of the study’s purpose, methods, risks, benefits, and right to withdraw at any time. Only after the participants voluntarily sign the written informed consent form will they be included in the study. For participants with limited decision-making ability, informed consent will be obtained from their legal guardians.

#### Privacy and Confidentiality

All participant information will be anonymized and coded. The original data (including informed consent forms, case report forms [CRFs], etc) will be stored in a locked file cabinet, and electronic data will be stored in a password-protected computer. Only authorized researchers can access the data. During data analysis and publication, personal identifying information will not be disclosed.

#### Participant Compensation

Participants will receive free electroacupuncture treatment (or sham electroacupuncture treatment) and related examinations (such as actigraphy monitoring) during the study period. In addition, to compensate for the time and travel costs of participants, each participant will receive a subsidy of 100 yuan (6.70 yuan=US $1, representing the consensus average forecast for the study period, ie, mid‑2026 to December 31, 2027) after completing the entire study (including treatment and follow-up).

### Patients and Participants

#### Overview

We will inform the participants of the study protocol and obtain written informed consent before screening them according to the eligibility criteria. The included participants will be randomly allocated at 1:1 to receive 4-week electroacupuncture treatment (electroacupuncture group) or sham electroacupuncture treatment (sham electroacupuncture group). We will then conduct an 8-week follow-up for further observation. All procedures will rigorously adhere to the Declaration of Helsinki. The inclusion and exclusion criteria are described below.

#### Inclusion Criteria

First, male or female participants who were aged 18 years or older. This age limit is set because adults have relatively stable physiological functions and can better cooperate with the treatment and evaluation process.

Second, patients diagnosed with SM based on cytological or histopathological features, and undergone SM surgery within 1‐2 weeks. This time interval is determined because sleep disturbance after SM surgery is most prominent within 1‐2 weeks postoperatively, and intervention during this period is more conducive to improving rehabilitation outcomes.

Third, the PSQI score was >11. Patients with chronic insomnia disorder (defined as insomnia lasting ≥3 months before surgery) are excluded. According to the validation study of the Chinese version of PSQI, a score >11 indicates moderate to severe insomnia, which is a reasonable threshold for selecting patients with clinically significant postoperative insomnia [31,32].

Fourth, have an estimated survival period of at least 6 months. This is to ensure that participants can complete the entire treatment and follow-up process, and avoid the impact of patient death on the study results. The survival period is estimated by the attending physician based on the patient’s tumor type, stage, treatment response, and other factors.

Fifth, consent to participation and completion of the full treatment process, and provide written consent.

Sixth, postoperative blood examination showed no obvious abnormality, indicating that the patient’s general condition is stable and can tolerate electroacupuncture treatment.

Finally, no history of shift work in the past 6 months. Shift work can independently affect sleep patterns and confound the study results, so such individuals are excluded.

#### Exclusion Criteria

First, participants diagnosed with severe cardiovascular, hepatic, renal, hematopoietic, immune system diseases, or psychiatric disorders. These diseases may affect the safety of electroacupuncture treatment or interfere with the evaluation of study outcomes.

Second, those who have a history of other malignant neoplasms or concurrent other malignant tumors. Multiple tumors may lead to complex clinical symptoms and poor prognosis, which may confound the effect of electroacupuncture on insomnia.

Third, participants’ acupuncture area affected with skin infection, ulcer, and sores. These skin conditions may increase the risk of infection after acupuncture and affect the implementation of treatment.

Fourth, those who have mental disorders or sequelae of cerebral infarction leading to inability to take care of oneself or cooperate with treatment. Participants need to cooperate with acupuncturists during treatment and complete self-assessment scales, so good compliance is required.

Fifth, those who have consciousness, intelligence, language comprehension, and expression impairments. These impairments will affect the collection of outcome indicators (such as self-assessment scales).

Sixth, those who have sleep apnea syndrome. This disease is an independent cause of sleep disturbance, which may interfere with the evaluation of the efficacy of electroacupuncture on postoperative insomnia.

Seventh, those who have taken any sleeping pills, antidepressants, or antipsychotics within the past 2 weeks. These drugs may affect sleep status and lead to false results in efficacy evaluation.

Eighth, history of shift work in the past 6 months.

Finally, participants having chronic insomnia disorder (insomnia lasting ≥3 months) before SM surgery.

### Recruitment

The study will include 196 patients with insomnia. We will recruit patients through offline promotion and the hospitals’ official information platforms. Researchers will maintain contact with interested patients and conduct face-to-face interviews to gain a deeper understanding of their characteristics and specific situations. To enroll in the trial, participants need to be informed of the benefits and adverse reactions of this trial after being screened by the researchers in accordance with the inclusion and exclusion criteria. Participants have the right to withdraw from the study at any time.

### Sample Size Calculation

The sample size was estimated based on anticipated changes in PSQI scores. Based on previous trials among populations with CRI, the mean difference of PSQI between 2 groups is assumed to be 1.8 (SD 3.9) for both groups [28]. Through Power Analysis and Sample Size system (version 15.0.5) calculation, a sample size can provide 90% power to reject the null hypothesis with a significance level of 0.05 using a 2-sided 2-sample *t* test assuming equal variance. Considering the expected dropout rate of 20%, the final sample size is 196, with 98 patients in each group.

### Randomization and Allocation Concealment

The random allocation scheme for this trial will be generated by The Clinical Research Center of Shanghai Municipal Hospital of Traditional Chinese Medicine, Shanghai University of Traditional Chinese Medicine. We will use stratified block randomization with centers as a stratification factor for group inclusion. When blocks are used up, centers will inform the data management center for replenishment. Independent investigators will place the treatment assignment code in consecutively numbered opaque envelopes based on the order of patients’ visits. All participants will be informed that they have the same probability of being assigned to the electroacupuncture group or the sham electroacupuncture group. Participants and researchers will not know the grouping in advance. To ensure the reliability of the research, all researchers will strictly abide by the principle of departmental separation and receive standardized training before implementation.

### Blinding

This study adopts a single-blind (patient-assessor–blinded) method. Except for the acupuncturists, other personnel, including all participants, outcome assessors, data analysts, and statisticians, will be blinded to the patients’ grouping. To reduce communication and contact between patients, we will assign patients to receive treatment in different rooms. During the entire treatment period, patients should wear eye patches. Blind evaluation will be used by the evaluator to assess and record various observation indicators. To ensure the safety of the study data, we will set up a data safety monitoring committee in accordance with the guidance of the Data Safety Monitoring Board (DSMB). The data will ultimately be summarized by third-party blind statistical analysis.

### Intervention

All study participants will be randomly assigned to either the electroacupuncture group or the sham electroacupuncture group in a 1:1 ratio, receiving treatment 3 times per week for 4 weeks. At the same time, we will keep records of actigraphy for patients at week 0, week 1, week 2, week 3, and week 4. Allocation of participants will be conducted by a licensed acupuncturist with more than 2 years of clinical experience. Except for the acupuncturists, the study investigators, participants, outcome assessors, study staff, and statisticians will all be blinded to the allocation. Before treatment, we will perform skin sterilization for patients. If patients experience severe physical discomfort due to insomnia, we will prescribe appropriate medication based on their conditions. During the medication period, we will strictly record the name, dosage, and administration method. Relevant investigators will also record the medication taken by patients on a CRF.

### Electroacupuncture Treatment (Electroacupuncture Group)

**Figure 1.**
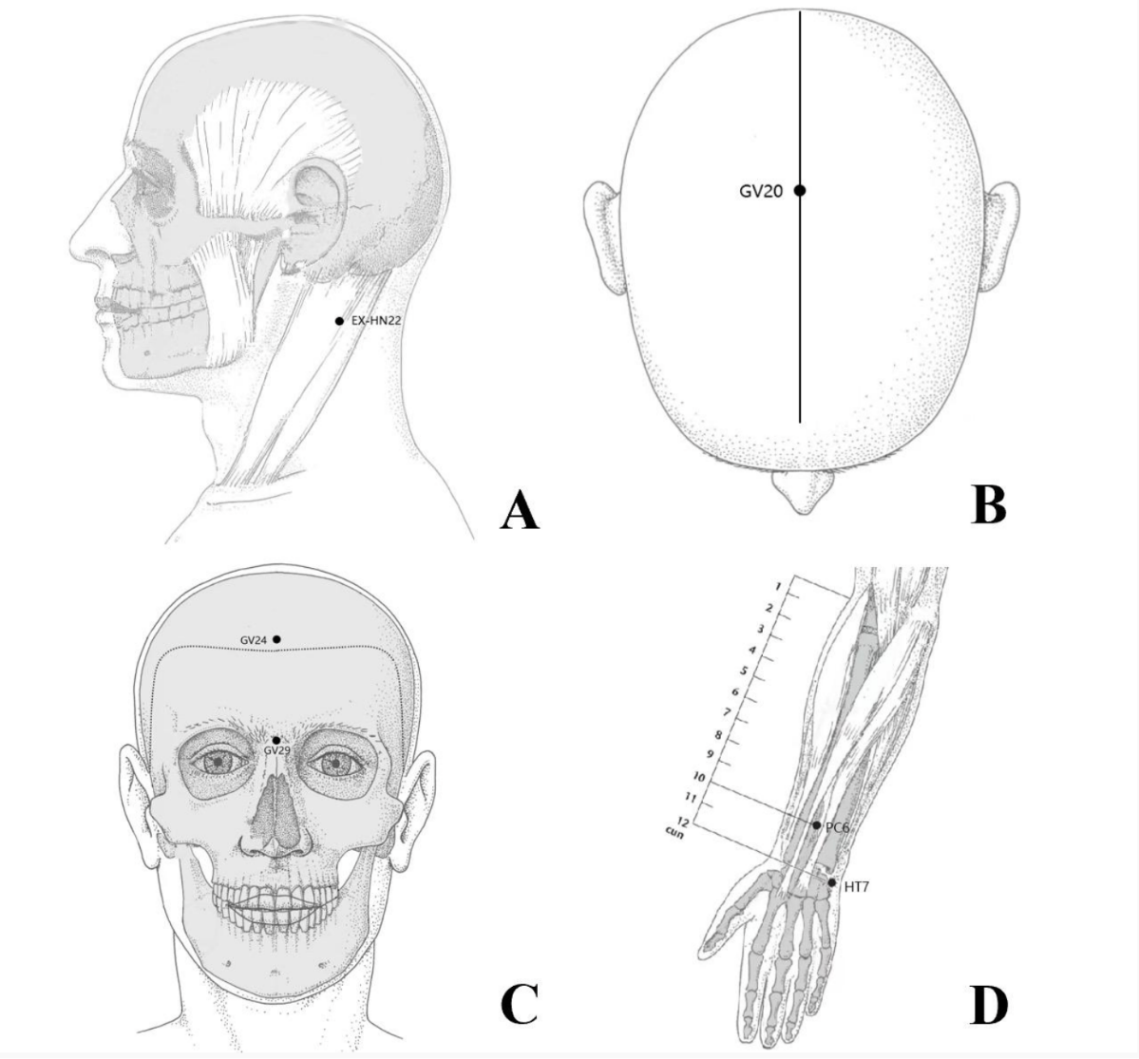
Location of acupoints. Bai Hui (GV20), Shen Ting (GV24), Yin Tang (GV29), An Mian (EX-HN22), Shen Men (HT7), and Nei Guan (PC6).

Patients in the electroacupuncture group will receive electroacupuncture performed by acupuncturists. Stainless steel 0.25×40 mm acupuncture needles (Suzhou Yan yi Medical Technology Co, Ltd) will be inserted into 6 acupoints, including Bai Hui (GV20), Shen Ting (GV24), Yin Tang (GV29), An Mian (EX-HN22), Shen Men (HT7), and Nei Guan (PC6) acupoints (Figure 1; for acupoint location, refer to The Location of Acupoints: State Standard of the People’s Republic of China [GB/T 12346‐2021] [33]). The locations, indications, and manipulation methods are specifically presented in Table 2. After the needle is inserted to a certain depth of the points, the acupuncturist will lift and thrust or rotate the needles to induce Deqi sensation. According to Traditional Chinese Medicine, there are 6 clinical manifestations of Deqi sensations, that are pain, numbness, distension, heaviness, dispersion, and dull pain [34,35]. After Deqi, the electroacupuncture device (CMNS6-1 [Wuxi Jia jian Medical Device Co, Ltd, China]) will be applied, connecting to points Bai Hui (GV20) and Yin Tang (GV29) with a frequency of 3 Hz, and the amplitude will vary depending on the patient’s comfort, limited between 2 and 5 mA to strengthen the needling sensation. The needle retention time is 30 minutes per session, as reported according to STRICTA guidelines.

**Table 2.** Locations and manipulations of the acupuncture group and sham acupuncture group.

Acupoints	Location	Methods
GV20 (Bai Hui)	5 cun directly above the midpoint of the posterior hairline, at the midpoint of the line connecting the apexes of the two auricles.	Subcutaneous insertion 16‐26 mm.
GV24 (Shen Ting)	0.5 cun directly above the midpoint of the anterior hairline.	Subcutaneous insertion 16‐26 mm.
GV29 (Yin Tang)	On the forehead, at the midpoint between the two medial ends of the eyebrow.	Subcutaneous insertion 16‐26 mm.
EX-HN22 (An Mian)	At the middle of sternocleidomastoid tendon.	Perpendicular insertion 33‐49 mm.
HT7 (Shen Men)	At the ulnar end of the transverse crease of the wrist, in the depression on the radial side of the tendon of musculus flexor carpi ulnaris.	Perpendicular insertion 10‐16 mm.
PC6 (Nei Guan)	On the palmar side of the forearm, 2 cun above the wrist crease, between the palmar longus tendon and the radial wrist flexor tendon.	Perpendicular or oblique insertion 16‐33 mm.

### Sham Electroacupuncture Treatment (Sham Electroacupuncture Group)

Patients in the sham electroacupuncture group will receive the sham electroacupuncture intervention treatment at the same acupoints as the treatment group. We will use the placebo needle named Streitberger placebo needle from Germany in this study [36]. It is blunt without a sharp tip and cannot penetrate the skin. This kind of needle can move inside the handle and appear to be shortened after puncturing without penetrating the skin. Therefore, it is difficult for patients to distinguish between sham acupuncture and real acupuncture. The electroacupuncture device will be connected to points Bai Hui (GV20) and Yin Tang (GV29) without any electrical current.

### Assessments and Outcomes Measurement

#### Primary Outcome

The PSQI will be the primary outcome of this trial. The cross-cultural adaptation of PSQI into simplified Chinese was successful, with good reliability and validity for comprehensively evaluating the sleep quality of patients with insomnia [31]. Furthermore, PSQI is widely used to assess the effect of cancer treatment on sleep quality in patients with cancer. PSQI comprises 19 self-assessment items and is composed of 7 subscales, including sleep quality, falling asleep time, sleep duration, sleep efficiency, sleep disorders, hypnotic drug use, and daytime dysfunction. Each component is scored on a scale of 0‐3, and the total scores of PSQI range from 0 to 21. The higher the patients’ score, the worse their sleep quality and the more severe the sleep disorders, and vice versa. The primary outcome is defined as the change in PSQI total score from baseline (wk 0) to posttreatment (wk 4).

#### Secondary Outcome

##### Records of Actigraphy

Actigraphy is a portable device worn on the wrist, capable of recording and analyzing physical activity patterns. As an objective measurement method, it is commonly used to evaluate sleep-wake patterns in sleep medicine. The American Academy of Sleep Medicine has demonstrated that actigraphy is an effective tool for assessing sleep disorders and circadian sleep-wake disorders [37].

**Figure 2.**
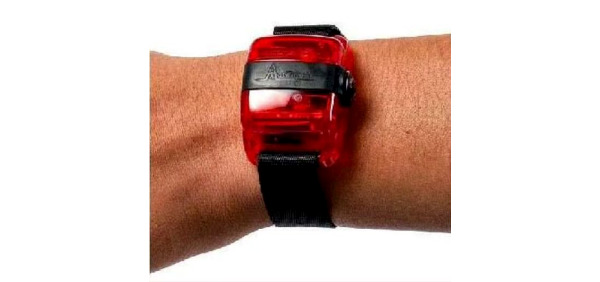
The actigraphy.

As a useful tool of sleep assessment, actigraphy is convenient, relatively low-cost, and easy to record patients’ activity for several weeks. By wearing it on the patient’s wrist, it can continuously record nighttime and daytime activity data, which can be used to analyze sleep and wakefulness patterns. Actigraphy is designed to estimate sleep latency (SOL), TST, sleep efficiency, wake after sleep onset (WASO), and the number of sleep awakenings of participants (Figure 2).

##### Insomnia Severity Index

Insomnia Severity Index (ISI) is a self-assessment scale specifically designed to evaluate the severity of insomnia. It shows good reliability, validity, and responsiveness, making it suitable for assessing sleep-related quality of life in China [38]. It has been widely used in clinical trials and research to measure the sleep quality of life of patients with cancer [39,40]. It consists of 7 items, including satisfaction of patients with insomnia with sleep quality, difficulty falling asleep, difficulty maintaining sleep, early awakening, the impact of sleep problems on daytime function, the level of concern or worry about insomnia issues, and the degree of distress caused by insomnia issues. With a total score range of 0‐28, the higher the score, the more severe the insomnia.

##### Spine Oncology Study Group Outcomes Questionnaire 2.0

The Spine Oncology Study Group Outcomes Questionnaire was developed by the Spine Oncology Study Group. In 2018, Versteeg et al [41] further validated the first version of this questionnaire and introduced the revised version, Spine Oncology Study Group Outcomes Questionnaire 2.0 (SOSGOQ 2.0). The revised version (SOSGOQ 2.0) has been extensively used in the literature to determine the effects of SM and its treatment [1,41,42]. Our term successfully verified the cross-cultural adaptation of SOSGOQ 2.0 into simplified Chinese, which demonstrated excellent acceptability, score distribution, internal consistency, test-retest reliability, and validity [4]. Therefore, the simplified Chinese version of the SOSGOQ 2.0 is considered a satisfactory tool for evaluating the health-related quality of life of Chinese patients with SM. The simplified Chinese version of the SOSGOQ 2.0 is composed of 6 health-related quality of life domains with 27 questions and 5 answer options, including physical function (6 items), neurological function (4 items), pain (5 items), mental health (2 items), social function (3 items), and posttherapy (7 items). Posttherapy questions will only be completed if the patient indicates having undergone post treatments for SM. Each question is answered via a 5-point Likert scale from 0 to 4. The overall score is calculated by summing the points of all items and excluding the posttherapy questions, with a maximum score of 80 and a minimum of 0. The higher the score, the worse the quality of life.

##### Patient Health Questionnaire-9

The Patient Health Questionnaire-9 (PHQ-9) is an effective tool for assessing the severity of depressive symptoms. The Chinese version of the depression scale, applicable to different populations, has high reliability and validity and can be used as a screening tool for evaluating the depression status of research participants [43]. PHQ-9 includes 9 items. The total scores range from 0 to 27—scores 0‐4 indicate no depressive symptoms, 5‐9 may indicate mild depressive symptoms, 10‐14 may indicate moderate depressive symptoms, 15‐19 may indicate moderate to severe depressive symptoms, and 20‐27 may indicate severe depressive symptoms.

### AEs

AEs related to electroacupuncture during this trial will be recorded in detail. A study provided information on AEs related to treatment, finding that AEs are all mild, including skin allergic reactions, hematomas, local pain, nausea, dizziness, and headache [44]. These AE data will be assessed in terms of severity and causality, and the incidence will also be determined. A 3-point grading system will be applied—grade 1 (mild), grade 2 (moderate), grade 3 (severe or medically significant). They will be recorded in the AEs form for further investigation by researchers who will analyze the causes of these events. The causality categories used will be certain, probable or likely, possible, unlikely, conditional or unclassified, and unassessable or unclassifiable. Serious AEs should be reported to the principal investigator (YC and MY) and Ethics Committee within 24 hours of occurrence.

### Assessment of Credibility

The Credibility Assessment Questionnaire is applied to assess the reliability and credibility of the trial, including the selection of the control group, the implementation of the blinding method, and the details of electroacupuncture treatment. The Credibility Assessment Questionnaire is used in electroacupuncture trials to improve the accuracy and repeatability of the trials. We will ask participants in this trial to rate the following questions on a 6-point scale: (1) How confident are you that this treatment can alleviate your complaints?; (2) If your friends also have similar complaints, how confident would you be in recommending this treatment method to them?; (3) How logical do you think this treatment is?; and (4) How successful do you think this treatment is in reducing other complaints? [45].

### Expectancy

After participants receive treatment, we expect that patients in the treatment group will have a significant improvement in sleep complaints compared with those in the sham electroacupuncture group, which will be reflected in the changes in outcome measurements. Electroacupuncture has shown certain positive effects on postoperative insomnia in patients with spinal cancer. It can be part of a comprehensive treatment plan in conjunction with other medications or nonpharmacological treatments to enhance therapeutic effects. All treatments should be carried out within a safe range to ensure the safety of electroacupuncture.

### Data Security and Monitoring

We will establish an independent DSMB to monitor whether the study design meets guideline standards to ensure the rigor of the trial. The committee consists of 5 members from different fields. Members of the DSMB must declare that there are no conflicts of interest to ensure the independence of their decisions. The DSMB will periodically review the accumulated data from clinical trials, assess the risks and benefits during the trial process, and ensure the safety of the patients. Based on the review of the data, the DSMB can advise the researchers to continue, modify, or suspend the trial. If serious AEs occur, they have the right to suspend or terminate the trial.

### Statistical Methods

#### Statistical Analysis Plan Documentation

A detailed statistical analysis plan (SAP) will be developed before data lock and unblinding, which will include additional technical details such as sample size justification, variable coding rules, and detailed analysis steps. The SAP will be signed by the principal investigator and statisticians (CJ, MY, XP, JZ, YC, FZ, JM, and DW) and will be available upon request from the journal or regulatory authorities.

#### Analysis of Primary and Secondary Outcomes (SPIRIT Item 20a)

##### Primary Outcome Analysis

The primary outcome is the change in PSQI total score from baseline (wk 0) to posttreatment (wk 4). ANCOVA will be used to compare the between-group difference in PSQI score changes, with baseline PSQI score, age, gender, tumor type, and participating center as covariates. This method can adjust for potential confounding factors and improve the statistical efficiency of the analysis.

##### Secondary Outcome Analysis

Actigraphy indicators (SOL, TST, sleep efficiency, WASO, and number of sleep awakenings): Between-group comparisons of changes from baseline to posttreatment (wk 4) and follow-up (wk 12) will be performed using ANCOVA, with corresponding baseline values and the same covariates as the primary outcome.

ISI, SOSGOQ 2.0, and PHQ-9 scores: Similar to the primary outcome, ANCOVA will be used to compare the between-group differences in score changes at each time point. If the data do not meet the normality assumption, nonparametric tests (Mann-Whitney *U* test for between-group comparisons and Wilcoxon signed-rank test for within-group comparisons) will be used as alternatives.

### Additional Analyses (SPIRIT Item 20b)

#### Subgroup Analysis

Predefined subgroup analyses will be conducted based on age ( ≤65 y vs >65 y), sex (male vs female), and tumor type (solid tumor vs hematological tumor) to explore whether the efficacy of electroacupuncture varies across different subgroups. Interaction terms between treatment group and subgroup variables will be added to the ANCOVA model to test for subgroup effects.

#### Sensitivity Analysis

To verify the robustness of the primary outcome results, sensitivity analyses will be performed by excluding participants who used hypnotic drugs during the study period and by using different imputation methods for missing data (eg, last observation carried forward vs multiple imputation [MI]).

### Analysis Populations and Missing Data Handling (SPIRIT Item 20c)

#### Analysis Populations

Intention-to-treat (ITT) population includes all randomized participants, regardless of protocol adherence, withdrawal, or missing data. This population will be used for the primary analysis to maintain the advantages of randomization and reduce selection bias.

Per-protocol population includes participants who completed the full course of treatment, had no major protocol violations, and provided complete outcome data. This population will be used for secondary analysis to evaluate the efficacy under ideal conditions.

Safety population includes all participants who received at least 1 session of treatment. AEs will be analyzed based on this population.

#### Missing Data Handling

MI with 20 imputed datasets will be used to handle missing outcome data in the ITT analysis. The MI model will include the primary outcome, secondary outcomes, baseline characteristics, and variables associated with missingness (eg, treatment group, participating center, and adherence to treatment). For the per-protocol analysis, only complete cases will be included.

### Statistical Software and Significance Level

All statistical analyses will be performed using SPSS 26.0 (IBM Corp) or R 4.2.0 software (R Core Team). The 2-sided significance level will be set at α=.05, and no adjustment for multiple comparisons will be made for secondary outcomes to avoid type II errors, but the results will be interpreted with caution.

## Results

This study is a prospective RCT that has completed the study design and obtained ethical approval. This study was funded in June 2023. It was supported by the Project of Shanghai Key Discipline Construction Project of Traditional Chinese Medicine (Clinical Category): Digital Intelligence Acupuncture (shzyyzdxk-2024108); the Project of Shanghai Municipal Health Commission (20204Y0165, 20224Y0165); the National Natural Science Foundation of China (82205145); Shanghai “Rising Stars of Medical Talents”-Youth Development Program-Youth Medical Talents-Specialist Program (SHWSRS 2023‐062); the Youth Talent Lifting Project of the Chinese Society of Traditional Chinese Medicine (2023-QNRC2-A03); and the “Visit Famous Schools and Worship Famous Teachers” Project of Shanghai University of Traditional Chinese Medicine (079). The funders had no role in the design of the study, data collection, analysis, interpretation of data, or writing of the manuscript.

Participant recruitment is projected to start in mid-2026 and end in December 2027, with a planned recruitment of 196 participants from 5 clinical institutions. As of the submission of this manuscript, the research team has completed the training of researchers, the preparation of study materials (such as informed consent forms, CRFs, and scales), and the debugging of equipment (electroacupuncture devices and actigraphy devices). The baseline characteristics of participants will be collected after recruitment starts, including age, sex, tumor type, surgical method, time since surgery, PSQI score, ISI score, etc

Data collection will be completed in October 2027, including baseline assessment (wk 0), treatment process records (weekly), posttreatment assessment (wk 4), and follow-up assessment (wk 12). Actigraphy monitoring will be conducted at week 0, week 1, week 2, week 3, and week 4 to collect objective sleep data. AEs will be recorded in real-time during the study period.

Data analysis is expected to be completed in December 2027. The primary analysis will focus on the difference in PSQI score changes between the electroacupuncture group and the sham electroacupuncture group based on the ITT population. Secondary analyses will include comparisons of actigraphy indicators, ISI score, SOSGOQ 2.0 score, and PHQ-9 score between the 2 groups at posttreatment and follow-up. Subgroup and sensitivity analyses will be performed to verify the robustness of the results. Safety analysis will evaluate the incidence and severity of AEs in both groups. The study results are anticipated to be published in the first half of 2028.

**Figure 3.**
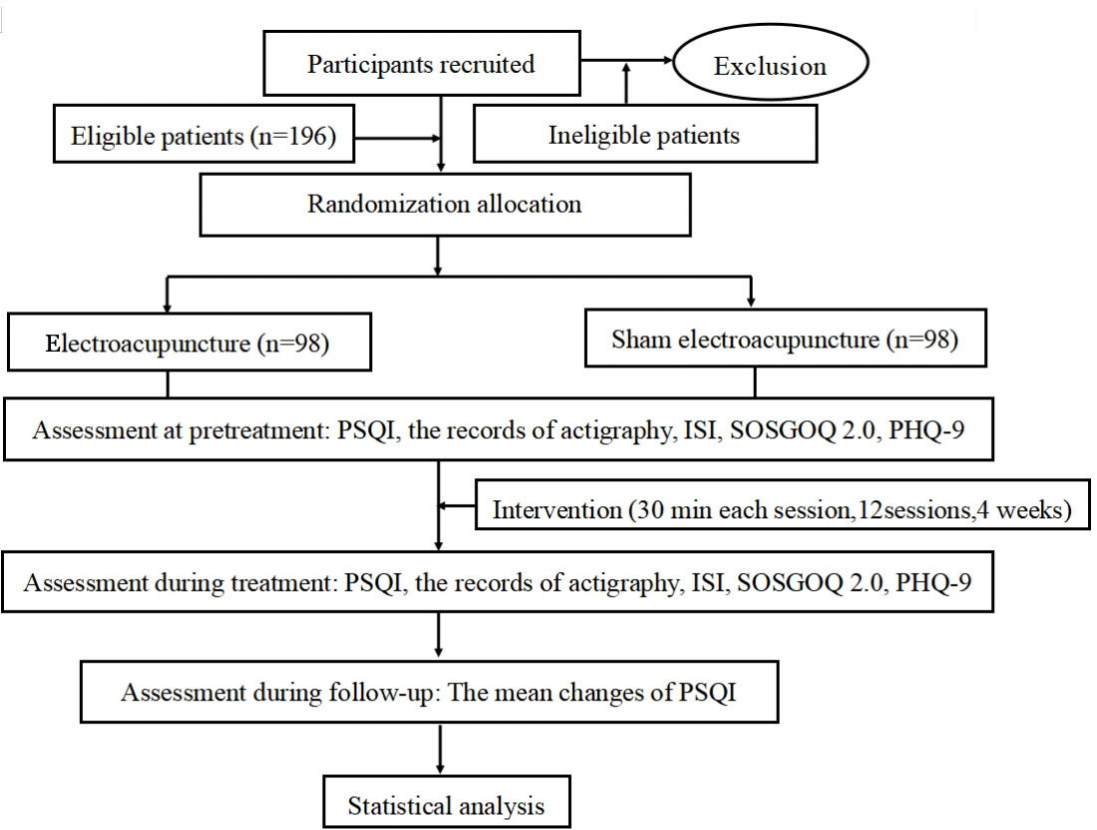
The trial flow chart. ISI: Insomnia Severity Index; PHQ-9: Patient Health Questionnaire-9; PSQI: Pittsburgh Sleep Quality Index; SOSGOQ 2.0: Spine Oncology Study Group Outcomes Questionnaire 2.0.

The trial flow chart is illustrated in Figure 3.

## Discussion

### Principal Findings (Anticipated)

This study is designed to test the hypothesis that compared with the sham electroacupuncture group, the electroacupuncture group will show a significant reduction in PSQI score from baseline to posttreatment (wk 4), indicating improved sleep quality. Based on previous evidence, electroacupuncture is expected to perform better in objective sleep indicators (such as increased sleep efficiency, prolonged TST, reduced number of sleep awakenings, shortened SOL, and decreased WASO), have a lower ISI score (reduced severity of insomnia), an improved SOSGOQ 2.0 score (enhanced quality of life), and a lower PHQ-9 score (alleviated depressive symptoms). In addition, electroacupuncture is expected to have a low incidence of AEs, demonstrating good safety.

### Comparison With Previous Work

Existing studies have confirmed the efficacy of electroacupuncture in the treatment of general insomnia and CRI. For example, a multicenter RCT by Lee et al [28] found that electroacupuncture can improve the sleep quality of patients with CRI. However, these studies did not specifically target patients with postoperative SM, who have unique clinical characteristics such as postoperative pain, spinal instability, and neurological damage. Patients with SM often have more complex sleep disturbance mechanisms, and the efficacy of electroacupuncture may differ from that in other populations.

In addition, most previous studies used small sample sizes and single-center designs, resulting in limited generalizability of the results. This study adopts a multicenter design, recruits 196 participants, and uses strict randomization, allocation concealment, and blinding methods to reduce bias, which is expected to provide more reliable clinical evidence. The selection of acupoints in this study is based on Traditional Chinese Medicine theory and clinical practice, and the treatment parameters (frequency, current intensity, and needle retention time) are standardized in accordance with STRICTA guidelines, facilitating the replication of the study results.

### Appropriateness of Sham Acupuncture Methodology

There has been extensive debate in the acupuncture research field regarding whether sham needling should be performed at true acupoints or nonacupoints. Proponents of nonacupoint sham needling argue that it can better control for the nonspecific effects of needle insertion, while advocates of true acupoint sham needling emphasize that the anatomical and physiological characteristics of acupoints may affect the blinding effect. In this study, we chose to use the Streitberger placebo needle at the same acupoints as the treatment group, and this choice is justified for the following reasons:

First, it ensures the success of blinding. The Streitberger placebo needle has a blunt tip that cannot penetrate the skin, but it can move within the handle, creating the illusion of needle insertion. For patients with postoperative SM who may have limited knowledge of acupuncture, this design makes it difficult to distinguish between real and sham acupuncture. If nonacupoints were used, the different anatomical locations might lead to differences in skin sensation, increasing the risk of unblinding.

Second, it controls for acupoint-specific effects. By using the same acupoints in both groups, the only difference between the electroacupuncture group and the sham electroacupuncture group is the presence or absence of effective needling (penetration and electrical stimulation), which can accurately evaluate the specific therapeutic effect of electroacupuncture. Previous studies have shown that true acupoint sham needling with placebo needles is an effective control method in acupuncture RCTs, as it can minimize the impact of nonspecific effects, such as expectation and attention [46,47].

Third, it is consistent with the clinical scenario. The acupoints selected in this study are commonly used in the clinical treatment of insomnia. Using the same acupoints for sham treatment ensures that the treatment process is similar to clinical practice, improving the external validity of the study. A systematic review by Xie et al [36] confirmed the validity of the Streitberger placebo needle in Chinese populations, further supporting the appropriateness of our choice. Therefore, the chosen sham electroacupuncture design is methodologically appropriate and consistent with contemporary recommendations for acupuncture RCTs.

### Strengths of the Study

This study has several strengths, such as (1) targeted population: focusing on patients with insomnia after SM surgery, a special population with unmet clinical needs, filling the gap in existing research; (2) rigorous study design: adopting a prospective, multicenter RCT design, with strict randomization, allocation concealment, and single-blind methods to ensure the internal validity of the study; (3) comprehensive outcome indicators: combining subjective (PSQI, ISI, etc) and objective (actigraphy) indicators to comprehensively evaluate sleep quality, and simultaneously assessing quality of life and depressive symptoms to reflect the overall therapeutic effect; (4) standardized intervention: the acupoint selection, manipulation methods, and treatment parameters of electroacupuncture are standardized in accordance with STRICTA guidelines, and acupuncturists are trained uniformly to ensure the consistency of treatment; (5) complete SAP: in accordance with SPIRIT guidelines, a detailed SAP is provided, including analysis methods for primary and secondary outcomes, additional analyses, and handling of missing data; and (6) complete safety monitoring: establishing a DSMB to monitor the study process, and recording AEs in detail to ensure the safety of participants.

### Limitations

Despite the rigorous design, this study still has some limitations. First, blinding limitations: although Streitberger placebo needles are used for the sham electroacupuncture group, acupuncturists are aware of the grouping, which may lead to performance bias. However, we have taken measures, such as separate treatment rooms and eye masks for patients to minimize this bias. Second, follow-up period: the follow-up period of this study is 8 weeks, which may not be sufficient to evaluate the long-term efficacy of electroacupuncture. Future studies can extend the follow-up period to observe the sustainability of the therapeutic effect. Third, lack of mechanism exploration: this study focuses on clinical efficacy and safety, and does not explore the underlying mechanism of electroacupuncture in improving sleep. Future studies can combine biological indicators (such as neurotransmitters and cytokines) to explore the mechanism. Fourth, exclusion criteria limitations: the study excludes patients with severe comorbidities, mental disorders, and chronic insomnia disorder, which may limit the generalizability of the results to the entire population with postoperative SM. Fifth, potential confounding factors: although we have adjusted for common confounding factors in the statistical analysis, there may still be unmeasured confounding factors (such as postoperative pain intensity and family support) that affect the study results. Finally, single-blind design: due to the characteristics of acupuncture treatment, it is difficult to implement double-blinding (acupuncturists cannot be blinded). This may introduce potential bias, but single-blinding of patients and outcome assessors can still reduce the impact of subjective factors.

### Future Directions

If the efficacy of electroacupuncture is confirmed in this study, future research can be carried out in the following directions: (1) optimize the treatment plan: explore the optimal acupoint combination, treatment frequency, and course of electroacupuncture for SM postoperative insomnia to further improve the therapeutic effect; (2) mechanism research: explore the mechanism of electroacupuncture in improving SM postoperative insomnia from the perspectives of neuroendocrinology, immunology, and circadian rhythm; (3) combined therapy: study the efficacy of electroacupuncture combined with other nonpharmacological therapies (such as cognitive behavioral therapy for insomnia and music therapy) in the treatment of SM postoperative insomnia; (4) expand the population: conduct studies in patients with SM with different tumor types, surgical methods, and disease stages to further verify the generalizability of the results; (5) long-term follow-up: extend the follow-up period to 6‐12 months to evaluate the long-term efficacy and safety of electroacupuncture; (6) individualized treatment: based on patient characteristics (such as age, gender, and tumor type), develop individualized electroacupuncture treatment plans to improve the precision of treatment.

### Dissemination Plan

The results of this study will be disseminated through multiple channels. First, academic conferences present the study results at domestic and international conferences on Traditional Chinese Medicine, oncology, and sleep medicine to exchange with peers. Second, academic journals publish the study results in high-quality peer-reviewed journals to provide evidence for clinical practice guidelines. Third, clinical promotions popularize the effective treatment plan among clinical doctors in relevant fields (such as orthopedics, oncology, rehabilitation, and Traditional Chinese Medicine) through training courses, workshops, etc, to benefit more patients with insomnia after SM surgery. Fourth, by public education, the research results are disseminated to the public through hospital official websites, WeChat (Tencent Holdings Limited) public accounts, and other platforms to improve the awareness of nonpharmacological therapies for insomnia. Finally, by providing policy recommendations to health administrative departments based on the study results, promoting the inclusion of electroacupuncture in the clinical management guidelines for SM postoperative insomnia.

This study will fill the methodological deficiencies of current clinical trials on acupuncture for the treatment of insomnia after SM surgery and provide a promising therapeutic intervention for clinical patients with insomnia after SM surgery.

## Abbreviations

AE: adverse event
CRF: case report form
CRI: cancer-related insomnia
DSMB: Data and Safety Monitoring Board
ISI: Insomnia Severity Index
ITT: intention-to-treat
MI: multiple imputation
PHQ-9: Patient Health Questionnaire-9
PSQI: Pittsburgh Sleep Quality Index
RCT: randomized controlled trial
SAP: statistical analysis plan
SM: spinal metastasis
SOL: sleep latency
SOSGOQ 2.0: Spine Oncology Study Group Outcomes Questionnaire 2.0
SPIRIT: Standard Protocol Items: Recommendations for Interventional Trials
STRICTA: Standards for Reporting Interventions in Clinical Trials of Acupuncture
TST: total sleep time
WASO: wake after sleep onset
